# Craniomaxillofacial Trauma in Dogs—Part II: Association Between Fracture Location, Morphology and Etiology

**DOI:** 10.3389/fvets.2020.00242

**Published:** 2020-05-15

**Authors:** Mercedes H. De Paolo, Boaz Arzi, Rachel E. Pollard, Philip H. Kass, Frank J. M. Verstraete

**Affiliations:** ^1^School of Veterinary Medicine, William R. Pritchard Veterinary Medical Teaching Hospital, University of California, Davis, Davis, CA, United States; ^2^Department of Surgical and Radiological Sciences, University of California, Davis, Davis, CA, United States; ^3^Department of Population Health and Reproduction, School of Veterinary Medicine, University of California, Davis, Davis, CA, United States

**Keywords:** craniomaxillofacial, trauma, computed tomography, fracture, displacement, dogs

## Abstract

Treatment of craniomaxillofacial (CMF) trauma in dogs requires a thorough understanding of the CMF skeletal structures involved. The human medical literature has several examples of CMF trauma and fracture classification, including the classically described Le Fort fractures. The recent classification schemes require large studies using computed tomography (CT). In the veterinary medical literature, such studies are lacking. The aims of part II of this retrospective study were to use a large number of CT studies of dogs evaluated for CMF trauma to determine whether specific fracture locations in the CMF region occur concurrently, and whether trauma etiology influences fracture morphology. This information may then be used to form a fracture classification scheme in the future. The medical records and CT studies of 165 dogs over a 10-year period were evaluated. The skeletal location of CMF fractures as well as the severity of displacement and fragmentation of each fracture was recorded. Dogs' demographic data and trauma etiology were also recorded. Fractured portions of the mandible tended to occur with fractures of adjacent bones, with the major exception of symphyseal separation, which occurred simultaneously with fractures of the cribriform plate. Fractures of the maxillary bone were accompanied by many concurrent fractures affecting the majority of the midface, skull base, and cranial vault. When the zygomatic bone was fractured, the other bones comprising the orbit also tended to fracture. Fractures of the relatively superficially located frontal and nasal bones were often accompanied by fractures of the skull base. Fracture etiology influenced fracture morphology such that vehicular trauma resulted in a relatively higher number of severely displaced and comminuted fractures than did other trauma etiologies. This study provides examples of fractures that, when found, should prompt veterinarians to look for additional injuries in specific locations. In addition, it further highlights the need for thorough CT evaluation of the entire CMF region, even when clinically apparent fractures appear relatively superficial.

## Introduction

Over a century has passed since the studies performed by Rene Le Fort in 1901, who demonstrated that fracture morphology and location are often closely related to trauma etiology (1). As in the human craniomaxillofacial (CMF) skeleton (2), the bones and anatomy of the canine CMF skeleton have many complex structures and interdigitations. Given this complexity, it is likely that neighboring bones will be fractured simultaneously as a result of trauma. What remains elusive is whether fractures of specific bones, such as the rostral mandible, are likely to occur simultaneously with more distant structures, for example the temporomandibular joint (TMJ). In people, there are specific fractures that, when found, should prompt clinicians to evaluate for further injuries (3). For example, fracture of the pterygoid bones is a feature of all Le Fort fractures and, when noted, should immediately prompt the clinician to look for evidence of additional fractures. Currently no such indicators exist for dogs that have sustained CMF trauma, and the potential usefulness of such indicators is evident.

Le Fort's studies were some of the earliest attempts at understanding how fracture etiology can affect the resultant fracture distribution and morphology (1). Since then, others have built upon his work and further refined it such that there are now different fracture patterns expected between and even within trauma etiology. For example, within ballistic injuries, different patterns are expected dependent upon weapon type and bullet caliber (4). Recently, it has been recognized that patterns of CMF trauma in people are likely to shift over time given that the nature of the trauma etiology (due to increased access to motor vehicles and weapons) itself is also shifting (2, 3, 5). In dogs, it is also intuitive that different trauma etiologies might result in different fracture patterns and severity. However, to the authors' knowledge this has not been documented. As we demonstrated in Part I, fracture location does tend to change based on etiology, but the resultant fracture morphology has yet to be reported.

An adequate understanding of these variables may provide a foundation for a fracture classification system in which trauma etiology; fracture location and morphology; and patient demographic factors are taken into account to inform prognosis and best practices. As described by Audigé et al. in the most recent AOCMF (Arbeitsgemeinschaft fur Osteosynthesefragen—craniomaxillofacial) trauma classification for humans (6), which has recently been validated (7), this process takes many iterations and requires collaboration between multiple specialists, and the first step requires documentation of the existing fractures and patterns.

At present, there is no evidence-based classification system for CMF fractures in the dog. An effective classification system for traumatic dentoalveolar injuries (TDI) in humans has recently been applied to TDI in dogs and cats with success (8). However, given the marked differences in CMF structure between humans and companion animals, no such classification system exists in the human literature that can be applied to CMF fractures in dogs. Therefore, an iterative process similar to that currently being undertaken by AOCMF will likely be needed in the future to produce a classification system that allows for appropriate communication across surgical specialties and, therefore, appropriate treatment of the dog as a whole.

In Part I, we demonstrated that trauma etiology is associated with fracture location (9). Similarly, fracture morphology can also vary based on the location of the fracture. In Part II, we elucidate whether certain bones or regions tend to fracture concurrently and whether there is a relationship between fracture etiology and fracture morphology. We hypothesized that specific bones or regions would fracture concurrently with others, and that fracture etiology would impact the resultant fracture morphology for each location differently.

## Methods

All methods relating to case selection, image acquisition, fracture evaluation, and categorization of demographic and trauma-related data were previously described in Part I of this study and are repeated below. All figures referenced in this Methods section appear in Part I of the accompanying paper (9).

### Case Selection

The electronic medical record database was queried for dogs that had been presented to the UC Davis Veterinary Medical Teaching Hospital for evaluation and treatment following CMF trauma between the years 2008–2018. All dogs had undergone computed tomography (conventional and/or cone-beam CT [CBCT]) at the initial visit. Exclusion criteria were as follows: trauma that occurred >1 week prior to presentation, dogs with CT scan slice thickness of >1.3 mm, and dogs for whom either the medical record or CT study were incomplete (e.g., the caudal-most portion of the skull had been left out of the study). Cases were excluded if the trauma occurred >7 days prior to presentation due to concern that: (a) early signs of fracture repair and boney remodeling may make fracture identification more difficult, and (b) further displacement may have occurred since the trauma. Exclusion of cases if the slice thickness was >1.3 mm was chosen as a compromise between maximizing the number of cases that were included in the study while simultaneously ensuring that slice thickness was not so large that small or incomplete fractures could be missed.

### Image Acquisition and Evaluation

All dogs received conventional (HiSpeed FX/i or LightSpeed16, GE Healthcare, Waukesha, WI) and/or cone beam CT (NewTom 5G CBCT Scanner, NewTom, Verona, Italy) scans at their initial visit. Although many dogs presenting for CMF trauma at our institution undergo CBCT, including conventional CT allowed the study to capture those cases in which superior soft tissue imaging was medically necessary (i.e., those with concern for intracranial hemorrhage, those too large for the CBCT field of view, and those who received treatment prior to the advent of CBCT at this facility). All DICOM files from each study were viewed using specialized software (Invivo5, Anatomage, San Jose, CA). Each case was viewed dynamically on medical flat-grade monitors (ASUS PB278Q 27-inch, ASUSTeK Computer Inc., Taipei, Taiwan), allowing the observers to utilize all viewing modes and tools to best assess all fractures. One observer (MD) viewed all studies and recorded all data after a period of calibration with one experienced board-certified radiologist (RP) and 2 board-certified diplomates and AVDC-OMFS Fellows (FJV, BA). When there was uncertainty, the particular study was reviewed with the board-certified radiologist (RP).

### Fracture Evaluation

**Figure 1:**
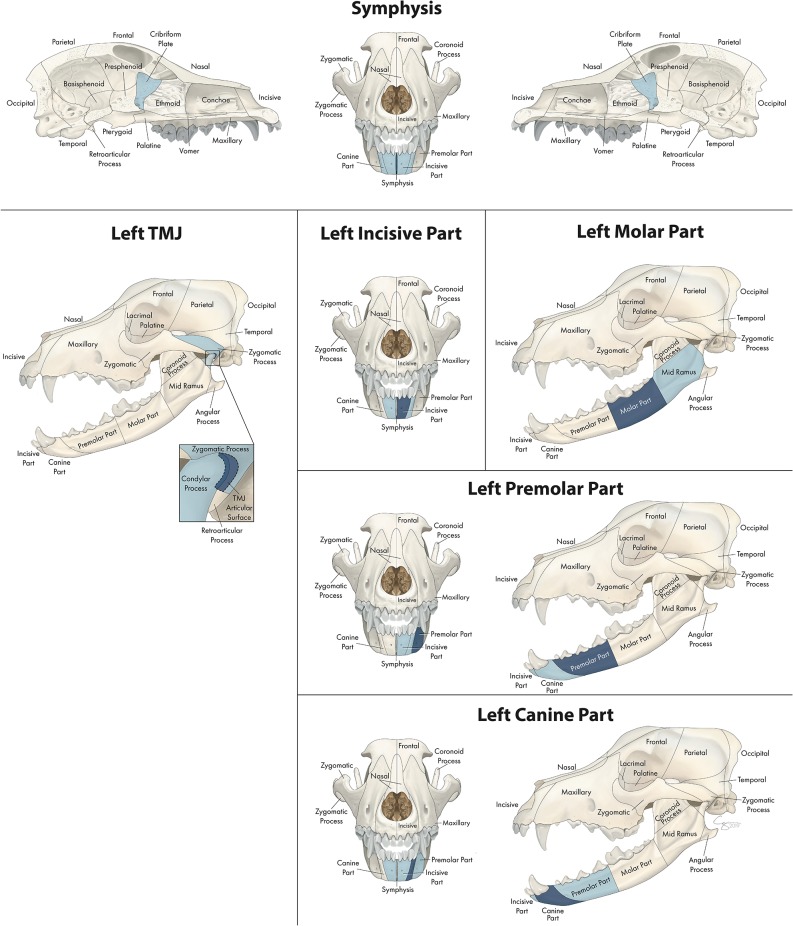
Significant bones or regions fractured concurrently with the various regions of the mandible. Although the left mandible is shown, when the right mandible was the region of interest, a mirror image of concurrently fractured locations applied. Only views of the skull that demonstrate a fractured region are shown.

Each skull was divided into specific bones and regions as illustrated in Part I, Figure 1. For each bone and region, it was determined whether each bone or region was fractured. If so, fracture morphology was described in terms of displacement and fragmentation. The degrees of displacement and fragmentation were modeled after the AOCMF fracture classification system (6). For both displacement and fragmentation, a score of 0 indicated no fracture. When scoring displacement, a score of 1 indicated no displacement, a score of 2 minimal displacement with ≥50% overlap remaining between fragments, and a score of 3 severe displacement with <50% overlap remaining. When scoring fragmentation, a score of 1 indicated an incomplete fracture, a score of 2 a complete fracture, and a score of 3 a comminuted fracture. This process was repeated on both the right and left sides of the skull. Although use of the term “comminuted” is discouraged by the most recent recommendations in human CMF literature (10), the term (and its associated meaning) are still pervasive in veterinary medicine and was therefore utilized in this study. A comminuted fracture was defined as a fracture having 3 or more bone fragments, although “minute” fragments were ignored unless the entire bone or region had been reduced to microfragments (11).

Because the bones that form the TMJ may be fractured without a fracture extending into the articular space, fractures of the temporomandibular joint were recorded separately from the condylar process, the retroarticular process, and the temporal bone. It was expected that there would be frequent overlap between these fractures. However, recording the instances of a fracture involving the articular surface itself was considered important enough to be coded separately. Similarly, although the cribriform plate is technically considered part of the ethmoid bone (12), the possible prognostic implications of having breached the braincase were deemed important enough to record instances of cribriform fracture separately from other ethmoid fractures.

**Figure 2:**
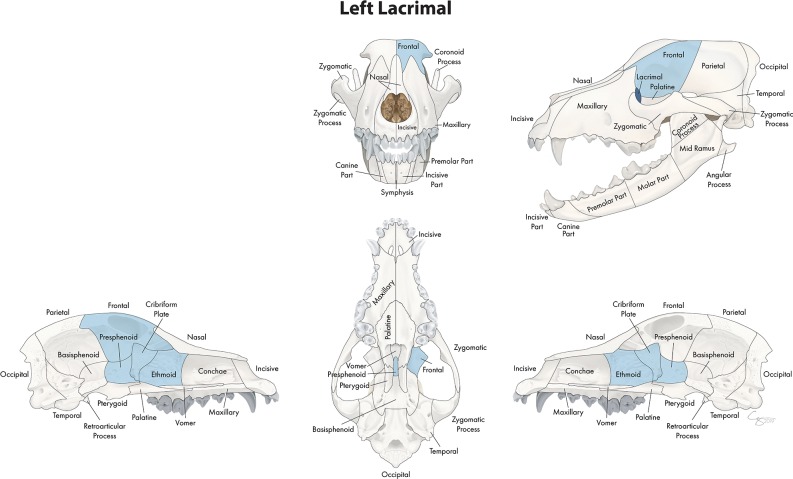
Significant bones or regions fractured concurrently with the lacrimal bone. When the lacrimal bone was the region of interest, the cribriform plate, ethmoid, presphenoid, and ipsilateral frontal bones fractured concurrently. Although the left lacrimal bone is shown, when the right lacrimal bone was the region of interest, a mirror image of concurrently fractured locations applied. Only views of the skull that demonstrate a fractured region are shown.

If a fracture occurred along a suture or at a border between two regions, the bone or region on both sides was considered fractured, and the morphology of the fracture was considered separately for each bone or region. By definition, all fractures along a suture were considered complete. However, the degree of displacement was recorded individually for the bone on either side of a suture (Part I, Figure 2).

**Figure 3:**
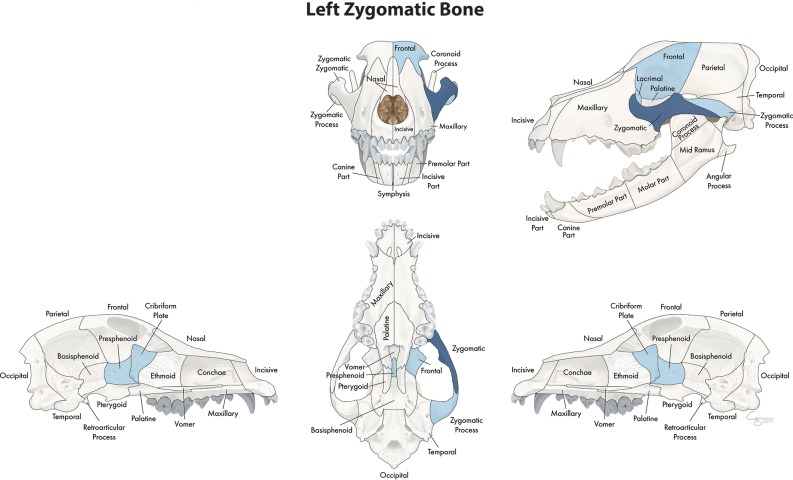
Significant bones or regions fractured concurrently with the zygomatic bone. When the zygomatic bone was the region of interest, the cribriform plate, presphenoid, and ipsilateral zygomatic process, lacrimal, and frontal bones fractured concurrently. Although the left zygomatic bone is shown, when the right zygomatic bone was the region of interest, a mirror image of concurrently fractured locations applied. Only views of the skull that demonstrate a fractured region are shown.

For the mandibular symphysis, a fibrocartilaginous joint (synchondrosis) symphyseal separation was considered by definition to be bilateral. However, if the 2 sides were unequally displaced such as depicted in Part I, Figure 3, the coding reflected this.

### Fracture Etiology

For each case, 1 of 7 different fracture etiologies were assigned, as depicted in Part I, Table 1.

### Statistical Methods

For each fracture location (the “region of interest”), exact binomial proportions and 95% confidence intervals were calculated to evaluate the frequency of related fractures occurring at other CMF locations. Locations with proportions >0.5 were reported only if the same association was detected on both sides of the jaw. For example, when evaluating maxillary fractures, the association was not reported if the left maxilla fractured concurrently with the right frontal bone but the right maxilla did not fracture concurrently with the left frontal bone. This was done to minimize the chance of reporting outlier associations. In addition, associations in which either the region of interest or the concurrently fractured location had fewer than 10 occurrences were not reported. When all of these criteria were met, the locations which fractured concurrently with the region of interest were recorded. These locations are henceforth referred to as “significant locations.”

Box-and-whisker plots were used to display the distribution of fragmentation and displacement severity scores at each potential fracture location. These analyses were conditional on each of 4 trauma type etiologies for which at least 20 cases were represented in the data.

## Results

Twenty bones or regions met inclusion criteria described earlier when assessing associations between fractured locations. Each of these associations is depicted in Figure 1 through Figure 12. The number of significant associations varied according to the primary fracture location being examined (the “region of interest,” which is depicted in dark blue), with some fracture locations only being associated with a single additional fracture location while others were associated with several additional locations (concurrently fractured locations highlighted in light blue). The bones or regions that met the criteria described above are grouped into 3 larger regions (the mandible, midface, and skull base/cranial vault) for further reporting of association.

### Fracture Location Co-occurrence: Mandible

#### Number of Concurrently Fractured Locations

In the mandible (Figure 1), the number of significant locations that fractured concurrently with the bone or region of interest ranged from one to five. When examining the molar part of the mandible, the only significant location that fractured concurrently was the mid-ramus region of the mandible. In contrast, when examining symphyseal separation, 5 regions fractured (or separated) concurrently including the contralateral and ipsilateral incisive and canine regions in addition to the cribriform plate.

#### Confinement to the Same Jaw

When the region or bone of interest was located in the mandible, the significant locations that were fractured concurrently were also located in the mandible, with two exceptions. The first exception was the mandibular symphysis, which was separated concurrently with fracture of the cribriform plate (along with fractures of both incisive and canine regions of the mandible). The second exception was the articular surface of the TMJ, which fractured concurrently with both the zygomatic process of the temporal bone as well as the condylar process of the mandible.

#### Distance of Concurrently Fractured Locations From Region of Interest

When examining different regions of the mandible as the primary location of interest, locations that fractured simultaneously tended to be adjacent to the region of interest and on the ipsilateral side. For example, the molar region tended to fracture simultaneously with the ipsilateral mid-ramus, and the premolar region fractured with the ipsilateral canine and molar regions. The instances in which the contralateral side of the mandible was fractured occurred only when the primary location of interest was in the rostral mandible (i.e., symphysis, canine, or incisive parts).

### Fracture Location Co-occurrence: Midface

#### Number of Concurrently Fractured Locations

**Figure 4:**
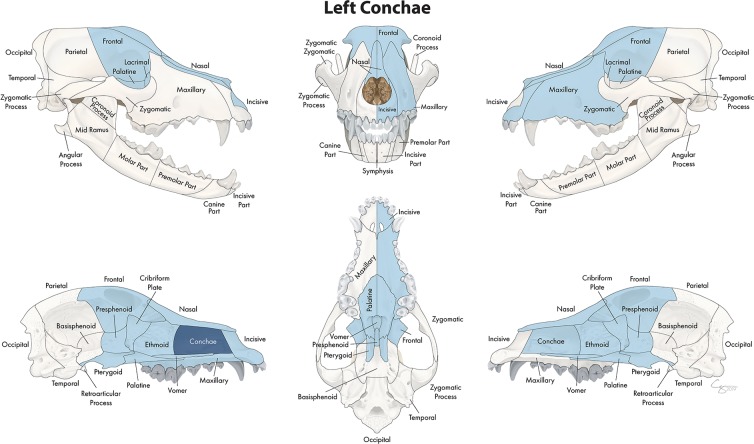
Significant bones or regions fractured concurrently with the conchae. When the conchae were the region of interest, many other bones of the skull base/cranial vault and midface, both on the ipsilateral and contralateral side, fractured concurrently. Although the left conchae are shown, when the right conchae were the region of interest, a mirror image of concurrently fractured locations applied.

**Figure 5:**
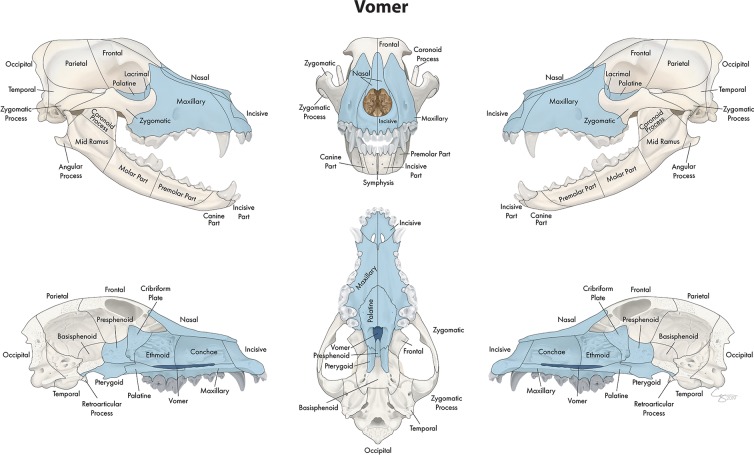
Significant bones or regions fractured concurrently with the vomer. When the vomer was the region of interest, the conchae, cribriform plate, ethmoid, presphenoid, and bilateral incisive, maxillary, nasal, lacrimal, palatine, and pterygoid bones fractured concurrently.

**Figure 6:**
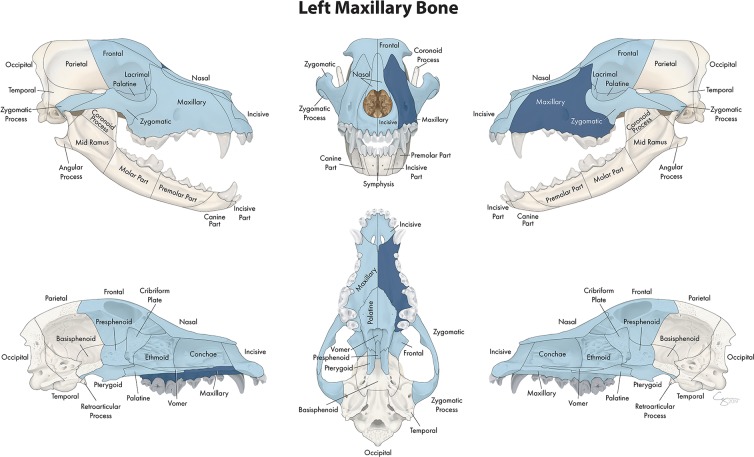
Significant bones or regions fractured concurrently with the maxillary bone. When the maxillary bone was the region of interest, many other bones of the skull base/cranial vault and midface, both on the ipsilateral and contralateral side, fractured concurrently. Although the left maxillary bone is shown, when the right maxillary bone was the region of interest, a mirror image of concurrently fractured locations applied.

**Figure 7:**
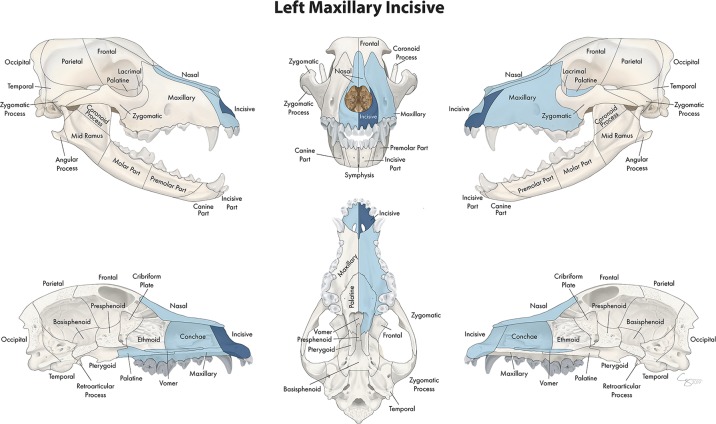
Significant bones or regions fractured concurrently with the incisive bone. When the incisive bone was the region of interest, the vomer, ipsilateral maxillary and palatine bones, contralateral incisive bone, and bilateral conchae and nasal bones fractured concurrently. Although the left incisive bone is shown, when the right incisive bone was the region of interest, a mirror image of concurrently fractured locations applied.

**Figure 8:**
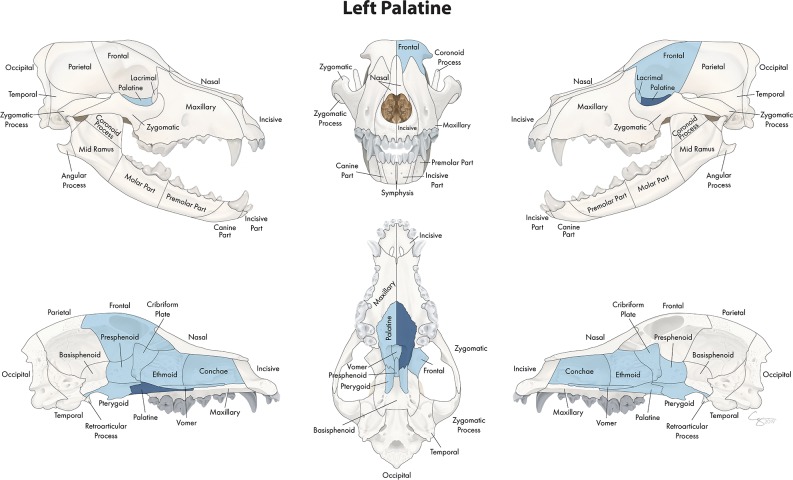
Significant bones or regions fractured concurrently with the palatine bone. When the palatine bone was the region of interest, the cribriform plate, vomer, conchae, ethmoid bone, presphenoid bone, contralateral palatine bone, bilateral pterygoid bones, and ipsilateral lacrimal and frontal bones fractured concurrently. Although the left palatine bone is shown, when the right palatine bone was the region of interest, a mirror image of concurrently fractured locations applied.

**Figure 9:**
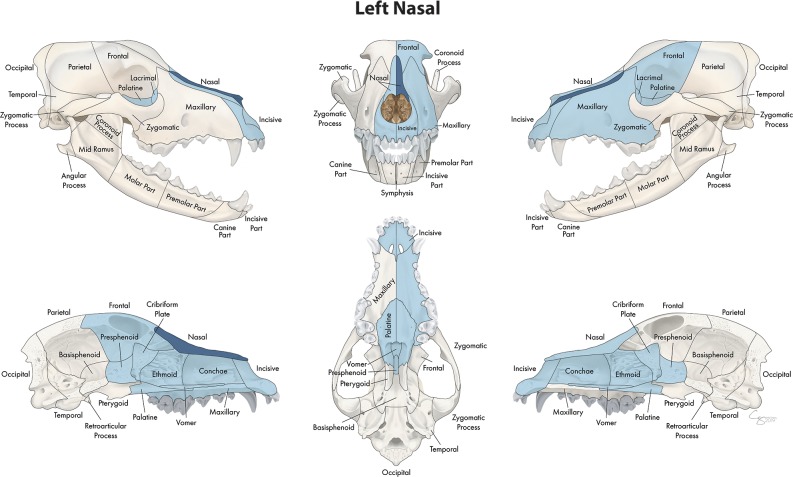
Significant bones or regions fractured concurrently with the nasal bone. When the nasal bone was the region of interest, many other bones of the skull base/cranial vault and midface, both on the ipsilateral and contralateral side, fractured concurrently. Although the left nasal bone is shown, when the right nasal bone was the region of interest, a mirror image of concurrently fractured locations applied.

In the midface, the number of locations that fractured concurrently with the bone or region of interest ranged from 4 to 23. When examining the lacrimal bone (Figure 2) or the zygomatic bone (Figure 3), for example, the number of significant locations that fractured concurrently was limited to 4 and 5, respectively. When the conchae (Figure 4), vomer (Figure 5), and maxillary bone (Figure 6) were isolated as the primary regions of interest, the number of significant locations that fractured concurrently was 17, 19, and 23, respectively. The number of locations that fractured with the incisive (Figure 7), palatine (Figure 8), and nasal bones (Figure 9) was intermediate, with 8, 11, and 15 concurrently fractured locations, respectively.

#### Confinement to the Same Jaw

When the region or bone of interest was located in the upper jaw, the significant locations that were fractured concurrently were also located in the upper jaw or the skull base and cranial vault.

#### Distance of Concurrently Fractured Locations From Region of Interest

When examining different bones and regions of the midface as the primary location of interest, locations that fractured simultaneously included those adjacent to the region of interest and on the ipsilateral side. However, a large number of fractures also occurred on the contralateral side and in regions not necessarily adjacent to the primary region of interest. For example, the conchae fractured with not only the surrounding regions but also many bones of the skull base and cranial vault. Similarly, the nasal bone fractured with multiple bones on the contralateral side as well as those of the skull base and cranial vault. The concurrently fractured bones and regions that occurred with fracture of the maxillary bone were the most broadly distributed, including the majority of the bones of the midface on the ipsilateral and contralateral sides of the skull. However, the zygomatic bone, conchae, incisive bone, lacrimal bone and vomer fractured concurrently with other bones and regions that were nearby.

### Fracture Location Co-occurrence: Skull Base and Cranial Vault

#### Number of Concurrently Fractured Locations

**Figure 10:**
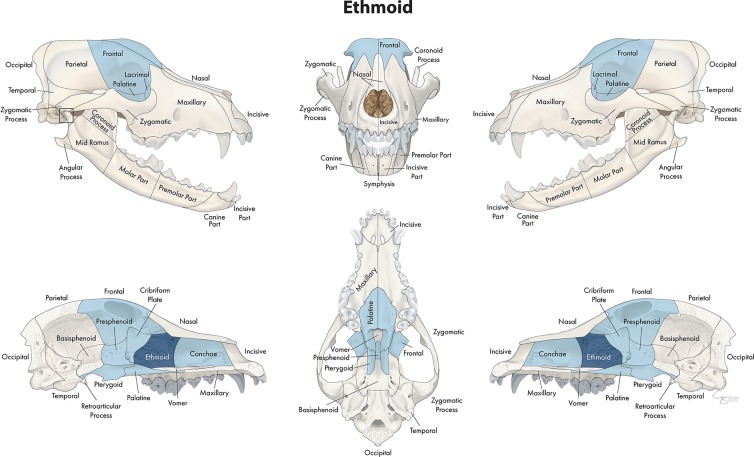
Significant bones or regions fractured concurrently with the ethmoid bone. When the ethmoid bone was the region of interest, the conchae, cribriform plate, presphenoid bone, and bilateral pterygoid, frontal, lacrimal, and palatine bones fractured concurrently.

**Figure 11:**
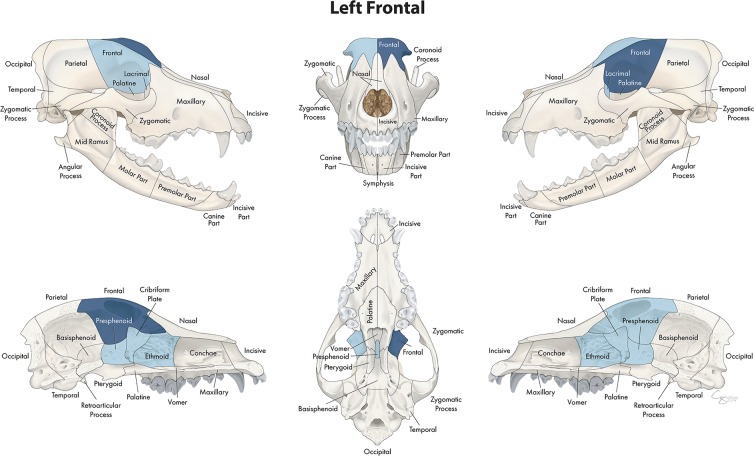
Significant bones or regions fractured concurrently with the frontal bone. When the frontal bone was the region of interest, the cribriform plate, ethmoid bone, presphenoid bone, ipsilateral lacrimal bone, and contralateral frontal bone fractured concurrently. Although the left frontal bone is shown, when the right frontal bone was the region of interest, a mirror image of concurrently fractured locations applied.

**Figure 12:**
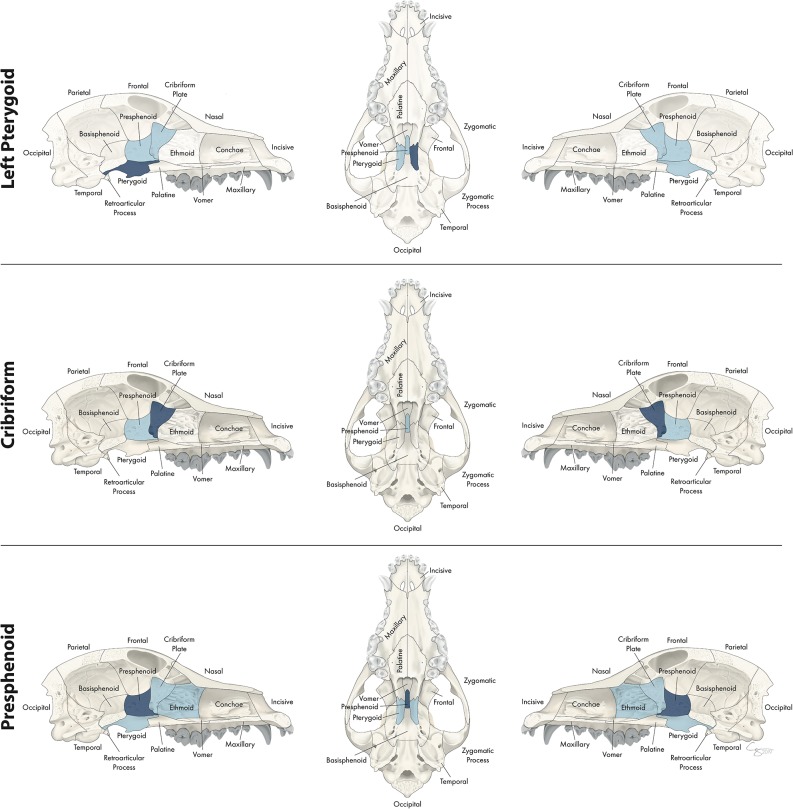
Significant bones or regions fractured concurrently with the pterygoid, cribriform plate, and presphenoid bone. When the pterygoid bone was the region of interest, the cribriform plate, presphenoid bone, and contralateral pterygoid bone fractured concurrently. When the cribriform plate was the region of interest, the presphenoid bone fractured concurrently. When the presphenoid bone was the region of interest, the cribriform plate, ethmoid bone, and bilateral pterygoid bones fractured concurrently. Although the left pterygoid bone is shown, when the right pterygoid bone was the region of interest, a mirror image of concurrently fractured locations applied. Only views of the skull that demonstrate a fractured region are shown.

In the skull base and cranial vault, the number of locations that fractured concurrently with the bone or region of interest ranged from 1 to 12. When examining fractures of the ethmoid bone (Figure 10) as a whole, 12 bones or regions fractured concurrently including frontal, palatine, pterygoid, and lacrimal bones bilaterally as well as the cribriform plate, presphenoid bone and conchae. The frontal bone (Figure 11) fractured concurrently with 5 other bones, and the presphenoid bone (Figure 12) with 4. When examining the pterygoid bones (Figure 12), they were found to fracture concurrently with the contralateral pterygoid bone as well as the cribriform plate and presphenoid bone. Whereas, when examining the cribriform plate (Figure 12), the only significant location that fractured concurrently was the presphenoid bone.

#### Distance of Concurrently Fractured Locations From Region of Interest

When examining different regions of the skull base and cranial vault as the primary location of interest, locations that fractured simultaneously tended to be adjacent to the region of interest. The exception to this finding was the ethmoid bone, which fractured concurrently with bones not just of the skull base and cranial vault but also with multiple bones of the midface such as the lacrimal and palatine bones.

### Fracture Etiology, Location, and Morphology

**Figure 13:**
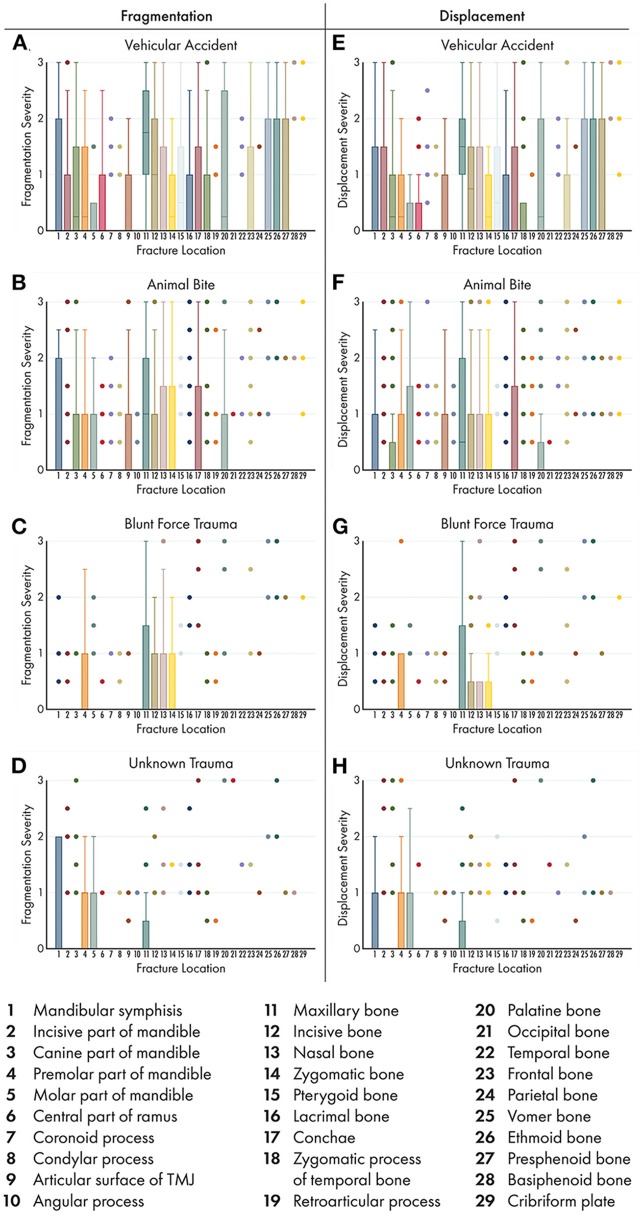
Box and whisker plots of fracture morphology by trauma etiology and fracture location. Box and whisker plots were used to demonstrate the severity of fragmentation **(A–D)** and displacement **(E–H)** of fractures occurring at each location based on trauma etiology. As in part I of this study, only those trauma etiologies that occurred in >20 cases were included for analysis. Boxes and whiskers represent the majority of the fractures recorded. Colored dots indicate outliers in the data. Therefore, locations with only colored dots visible indicate that the fragmentation and severity score was 0 (the locations were not fractured) the majority of the time. No attempt was made to determine significance in patterns across data.

Box and whisker plots demonstrate the severity of fragmentation and displacement of fractures occurring at each location (1-29) based on trauma etiology (Figure 13). As in Part I, only those trauma etiologies that occurred in more than 20 cases were included for analysis. Whiskers represent the fragmentation and displacement severity of the majority of the fractures recorded at that location, while the colored dots indicate outliers in the data. Therefore, locations with only colored dots visible means that the fragmentation and severity score was 0 (the locations were not fractured) the majority of the time with several outliers. No attempt was made to determine significance in patterns across data. A general finding across all trauma etiologies was that those fracture locations with higher fragmentation scores also had higher displacement scores.

#### Vehicular Accidents

The majority of fractured locations occurring secondary to vehicular accidents had low, but >0, fragmentation and displacement scores, although the whiskers on the box plot did span the entire range of (0–3) for several locations. The notable exception was the maxillary bone, in which the majority of cases had fragmentation and displacement scores closer to 2 (complete fracture and minimally displaced fracture, respectively). The majority of vehicular accident cases had fractures of the vomer, ethmoid, and presphenoid bones with whiskers spanning the entire severity score range, whereas no other trauma etiology displayed this pattern.

#### Animal Bites

Eleven of the 29 possible fracture locations affected by animal bites had fragmentation and displacement scores >0 in the majority of cases, whereas the remaining 18 locations were unaffected in the majority of cases. Again, the most exposed regions of the CMF skeleton (the mid to rostral mandibles and the midface) had higher severity scores in both fragmentation and displacement than did other less exposed regions.

#### Blunt Force Trauma

Only 5 of the 29 possible fracture locations affected by blunt force trauma had fragmentation and displacement scores >0 in the majority of cases, whereas the remaining 24 locations were unaffected in the majority of cases. Unlike for the other 3 trauma etiologies, the symphysis and the molar region of the mandible had fragmentation and displacement scores of 0 (they were unaffected) in the majority of cases.

#### Unknown Trauma

Only 4 of the 29 possible fracture locations affected by unknown trauma had fragmentation and displacement scores >0 in the majority of cases, whereas the remaining 25 locations were unaffected in the majority of cases. Unlike the other three trauma etiologies, the maxillary, incisive, nasal, and zygomatic bones had fragmentation and displacement scores close to 0 (they were unaffected) in the majority of cases.

## Discussion

This study examines CMF trauma in dogs using CT as a diagnostic tool and further details the relationships between fracture location, morphology, and etiology. We report several key findings. First, the use of CT was instrumental in determining that spatially distant bones or regions fractured concurrently. Second, the bone fractures that occurred concurrently with fractured regions of the mandible, the orbit, the nasal cavity, and the maxilla are likely to be of clinical and prognostic significance. Finally, trauma etiology is associated with fracture morphology. As a result, the hypothesis that specific bones or regions would fracture concurrently with others, and that fracture etiology would impact the resultant fracture morphology for each location differently is accepted.

We demonstrated that in dogs affected by CMF trauma, there are often multiple bone fractures regardless of the etiology. This further signifies the importance of CT for complete and accurate diagnosis. As has been reported previously (13), skull radiographs typically underdiagnose the presence of fractures in maxillofacial trauma. In turn, this may have a significant impact on treatment plan and prognosis. For example, symphyseal separation is readily apparent on physical examination, and a clinician may make the erroneous assumption that this is the only injury in the absence of a CT. Importantly, our study showed that in cases of symphyseal separation, the cribriform plate is also fractured, which is highly relevant for treatment recommendations and prognosis. Although many human CMF trauma patients with CSF leaks heal without additional surgical intervention (14, 15), intensive monitoring for meningitis and other sequelae should be considered. It is reasonable to assume that similar recommendations are warranted in dogs.

In the mandible, there were fracture locations that co-occur, which are likely to be clinically relevant for treatment planning. Specifically, the molar part of the mandible fractures with the ramus of the mandible, whereas the premolar part is more likely to fracture with the canine and incisive parts. This suggests that fractures of these two regions may require different fixation strategies. For example, fractures of the molar part of the mandible typically do not have enough substantial teeth caudal to the fracture line to support an interdental wire and composite splint (16). Therefore, internal fixation emerges as a better option. However, if the ramus of the mandible is also fractured, and especially if it is fractured in multiple locations, placing internal fixation is more challenging owing to the very thin nature of the bone and overlying soft tissue (17). In addition, even if identification of concurrently fractured regions does not result in an immediate change in fixation strategy, it may necessitate the need for future monitoring of tooth vitality given that the mandible is largely a tooth-bearing bone.

When examining the concurrently fractured locations with the zygomatic bone, it was apparent that the various skeletal structures comprising the orbit tend to be affected simultaneously. The zygoma, frontal bone, and lacrimal bone, which together form the majority of the orbit, (12), all tend to be affected. As is well-established in humans, reconstructing the orbital dimensions has significant clinical and cosmetic implications. While the cosmetic implications are not a primary goal in veterinary medicine, clinical implications such as diplopia, muscle entrapment, and impingement on neurovascular structures are clinically important. This underscores the need for thorough examination all of bones concurrently with appropriate imaging. Although dogs, lacking an orbital floor equivalent to that in humans, are less prone to muscle entrapment and similar clinical complications, fixation should nonetheless focus on retaining/reconstructing the orbit as much as possible. In addition, involving an ophthalmologist in the case when there are fractures of the orbit may be an important step in patient management.

Fractures of the conchae, vomer bone, and nasal bones tended to co-occur. Importantly, nasal bone fractures may be amenable to and may even require fixation to help reconstruct the nasal cavity to maximize airflow and prevent formation of a sequestrum or other complication (17). Although the conchae and vomer bones may not be surgically addressed when fractured, the presence of fractures in this region is important to diagnose and monitor, as it can predispose the patient to chronic rhinitis, fungal disease, or stenosis. In some cases, surgical exploration and stenting of the nasal cavity is indicated to prevent these complications (18, 19). Therefore, finding that the conchae and vomer tend to fracture concurrently with the nasal bone has clinical significance in that if a nasal bone fracture is identified, the underlying bones and deeper structures should be examined to optimize treatment.

As was demonstrated in Part I of this study, the maxillary bone was the most commonly fractured location regardless of trauma etiology. In this part of the study, we found that the maxillary bone also tends to fracture concurrently with the highest number of other bones or regions, including the midface, skull base, and cranial vault. Therefore, when a maxillary fracture is noted on physical examination or diagnostic imaging, this should prompt the clinician to thoroughly evaluate not only neighboring bones but also those in distant locations. Interestingly, the mandibles and maxillary bones did not tend to fracture concurrently in the majority of cases, but this should not preclude complete evaluation of both regions.

We found that trauma etiology is also associated with fracture morphology in dogs, which is consistent with the human medical literature (1, 20, 21). In our study, severely fragmented or displaced fractures were less common overall than those that were non-displaced. However, vehicular accidents, which often involve a higher velocity impact than the other trauma types examined, resulted in fractures with a higher degree of displacement and fragmentation. The potential importance of this for veterinarians lies in the need to properly visualize all fragments and their spatial relationship. Tridimensional (3D) imaging is especially important in these cases so that surgical planning can take into account the relative locations, sizes, and shapes of fracture fragments. In the human literature, it is well-accepted that 3D imaging (2, 22, 23) is superior for treatment planning related to CMF trauma and is commonly being used for intraoperative visualization as well (24).

The limitation of this study is inherent to its retrospective design. In addition, the dogs included in this study were assessed at a tertiary referral institution, which could have affected the types of CMF trauma included in the study. For example, very mild cases may not have been referred to our institution if the primary veterinarian felt capable of treating the patient. Likewise, very severe cases may have died or been euthanized prior to referral. Because several of the trauma etiologies (crush injuries, fall from height, and ballistic traumas) occurred infrequently, the sample size for those etiologies was too small to draw any conclusions from the associated data. Although not specifically addressed in this study, documentation and treatment of dentoalveolar trauma is of clear importance when caring for any CMF trauma patient and has been thoroughly discussed elsewhere (8). A limitation of particular relevance to part II of this study is that skull conformation was not specifically included as a variable. For example, it is possible that concurrent fracture locations in brachycephalic dogs may be different than those in dolichocephalic dogs. However, precise determination of skull conformation requires measurements between fixed points in the CMF skeleton which are inherently disrupted and potentially distorted when fractured.

In conclusion, this study further elucidates the relationships of fracture etiology, location, and morphology in dogs that sustained CMF trauma. We highlight the importance of CT evaluation of the entire CMF region in CMF trauma in dogs. In addition, this research underscores the need for thorough systemic evaluation of CMF trauma cases to ensure that there are not more pressing concurrent injuries (e.g., traumatic brain injury) that require immediate treatment. Finally, we laid the foundation for future studies to address classification of CMF fractures and trauma. As with development of the human AOCMF classification system (6), developing a classification system in dogs will be an iterative process and will require multi-institutional cooperation for validation. Therefore, a classification system was not proposed based solely on the results of this study. However, it is important to note that although certain bones or regions do tend to fracture concurrently, there are a large number of bones which fracture independently, and this finding must be accounted for when developing possible classification systems.

## Data Availability Statement

The datasets generated for this study can be made available from the authors upon request.

## Ethics Statement

Ethical review and approval was not required for the animal study because the study is retrospective in nature and included clinical cases; hence, it is exempt from IACUC requirements. Written informed consent for participation was not obtained from the owners because the study is retrospective in nature and hence, it is exempt from written informed consent.

## Author Contributions

MD: study concept and design, image analysis, data acquisition, analysis, interpretation, drafting of the manuscript, and final approval of the version to be published. RP: study concept and design, image analysis, drafting of the manuscript, and final approval of the version to be published. BA and FV: study concept and design, data interpretation, drafting of the manuscript, and final approval of the version to be published. PK: data analysis and interpretation, drafting of the manuscript, and final approval of the version to be published.

## Conflict of Interest

The authors declare that the research was conducted in the absence of any commercial or financial relationships that could be construed as a potential conflict of interest.
